# An advanced multisystem histiocytic sarcoma in a pregnant woman: A case report

**DOI:** 10.1016/j.radcr.2024.05.069

**Published:** 2024-06-21

**Authors:** Amirhossein Soltani, Mohsen Salimi, Mahdi Saeedi-Moghadam

**Affiliations:** a Department of Radiology, Shiraz University of Medical Sciences, Shiraz, Iran; b Medical Imaging Research Center, Shiraz University of Medical Sciences, Shiraz, Iran; c Student Research Committee, School of Medicine, Shiraz University of Medical Sciences, Shiraz, Iran

**Keywords:** Histiocytic sarcoma, Pregnant, Chest X-rays, Chest CT, Extranodal histiocytic sarcoma

## Abstract

Histiocytic sarcoma is an extremely rare disease that's hard to diagnose and treat, often leading to a poor prognosis. Here, we present a case report detailing a rare occurrence of HS in a 37-year-old pregnant woman who first presented with left shoulder pain, palpitations, and a productive cough at 20 weeks of gestation. Her diagnostic evaluations were performed, including different imaging modalities such as chest X-rays, CT scans, and MRI. Imaging revealed a large mediastinal mass with extensive involvement of the adrenal glands, lungs, and lymph nodes. The definitive diagnosis of HS is based on pathological and morphological features, and the immunohistochemistry report plays a key role. In our case, the diagnosis of HS was confirmed through pathological evaluation and immunohistochemistry, with a positive CD68 result obtained from a supraclavicular lymph node biopsy. A hospital committee comprising medical specialists like hematologists-oncologists, pathologists, pulmonologists, and obstetricians was brought together to assess the case collectively. The patient received chemotherapy, which alleviated her symptoms and maintained her condition. Based on the committee's recommendations, despite a healthy fetus and normal obstetric sonograms, the decision was made to terminate the pregnancy with the consent of the patient and her family. Despite initial improvement postchemotherapy, the patient's condition worsened, necessitating intubation. Tragically, two months after the initial admission, the patient passed away due to severe complications. In this case report, we provide a literature review and review of the patient's imaging reports. Since the patient is pregnant and HS is uncommon, it's important to highlight that this case is unique and worth sharing.

## Introduction

HS is extremely rare, especially in the context of pregnancy. It is challenging in terms of diagnosis and treatment, often leading to a poor prognosis [1].

While the diagnosis of histiocytic sarcoma (HS) primarily relies on its morphological and pathological characteristics, with immunohistochemistry (IHC) playing a key role, imaging and radiologic features are particularly valuable, especially when dealing with cases of extranodal HS occurrence [2].

Here, we present a case of pathologically proven histiocytic sarcoma in a pregnant woman who developed a large mediastinal mass. Taking a step further, we discuss certain radiologic features that aided in the disease diagnosis.

Despite several cases of HS documented in medical literature, The occurrence of HS as a mediastinal mass during pregnancy is very rare and hasn't been extensively addressed in medical literature, particularly regarding how to diagnose and treat it. The complexity of this case and the decision-making steps following a diagnosis challenge could help healthcare providers face similar presentations in the future.

A 37-year-old pregnant woman with no significant past medical history presented with left shoulder pain, palpitation, and productive cough at a Gestational Age of 20 weeks. She was referred to a pulmonologist, who requested an ECG and spirometry test, the results returned normal, revealing no significant findings. One week later, the patient's symptoms were exacerbated, and she developed chest tightness and tachypnea in addition to the symptoms she had previously. She was directed to a hospital; her initial lab results indicated a high white blood cell count (WBC = 31,000/µL) and elevated C-reactive protein (CRP = 143 Mg/L).

Due to a strong clinical suspicion of pulmonary thromboembolism (PTE) and her pregnancy, a lung perfusion scan was performed for her. The results ruled out PTE and showed significant diffuse hypoperfusion in the left lung, suggesting the presence of other lesions rather than PTE.

**Fig. 1:**
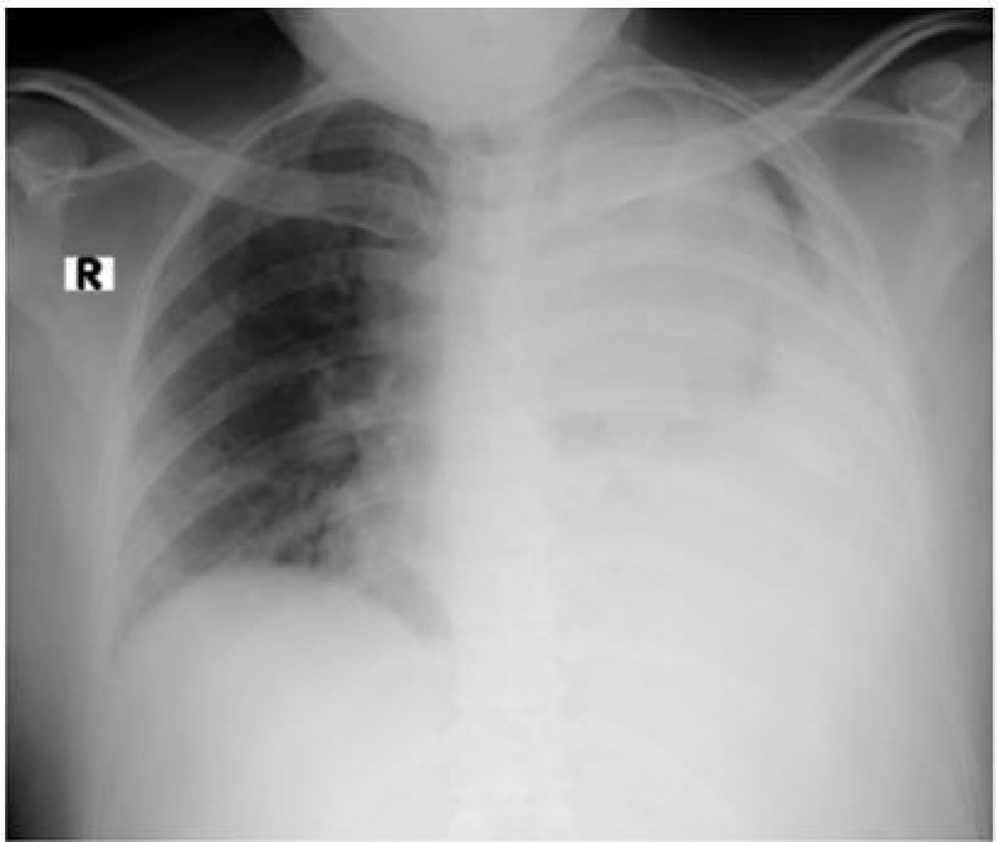
CXR shows left side white lung with blunting of the costophrenic angle and with no sign of significant heart and mediastinal shift.

**Fig. 2:**
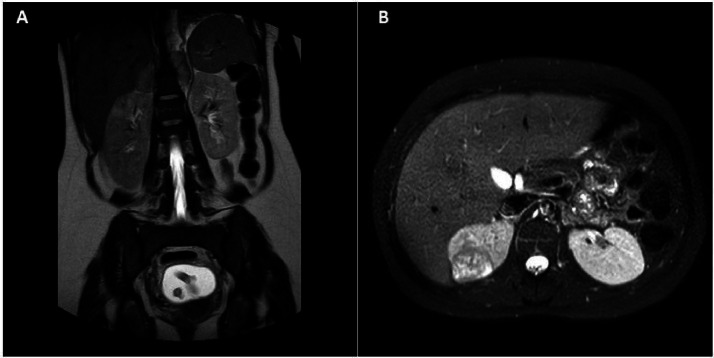
(A) Coronal view T2W Sequence shows Bilateral Adrenal Gland tumor. Also, in the visualized part of the uterus, the fetus' limbs are seen in the gestational sac in the endometrial cavity. (B) The T2W-SPAIR Axial view shows well-defined heterogeneous high.

The patient was transferred to a center with ICU capabilities for more evaluations (Namazi Hospital). During the initial procedures, a chest X-ray was performed for the patient, revealing a left-sided white lung (Fig. 1). Also, an abdominal Ultrasound suggested a mass lesion at the suprarenal area in favor of adrenal mass. For further evaluation, an abdomen and chest MRI was done, and heterogeneous iso-intense lesions were found in the upper pole of the right Kidney and both Adrenal glands (Fig. 2).

**Fig. 3:**
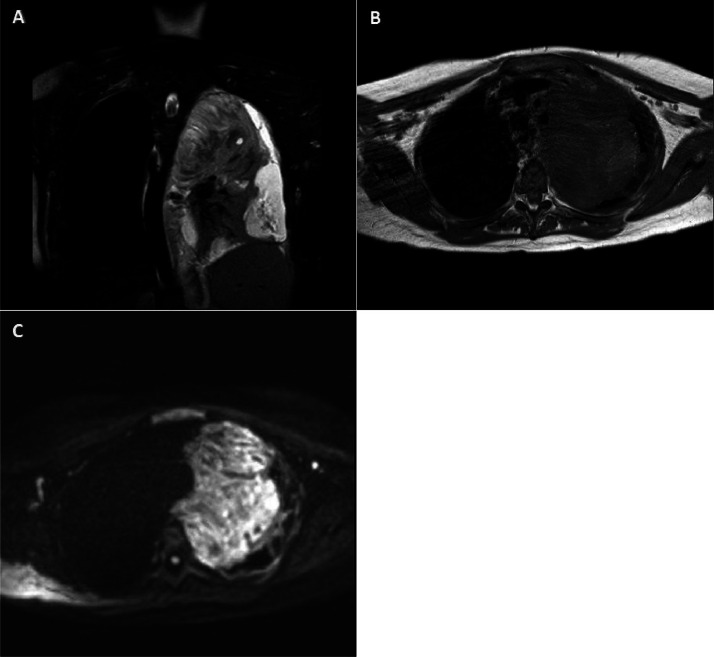
(A) Coronal T2W-SPAIR, (B) axial T1W, and (C). axial DWI sequences show large lobulated Mass with high T2W and iso to low T1W signal intensity measuring 140 × 100 × 95 mm with diffusion restriction in the Left side of the mediastinum with extension to the left hilum and upper and lower lobe.

In Chest MRI, a lobulated mass with diffusion restriction was seen in the mediastinum, with extension to the left hilar region and upper and lower lung lobes. Multiple nodules were detected in different lobes of the right lung, and there were multiple prominent lymph nodes, especially the large necrotic one in the right supraclavicular area. Additionally, thickening and irregularity of the left pleura raise concern for possible metastasis (Fig. 3).

**Fig. 4:**
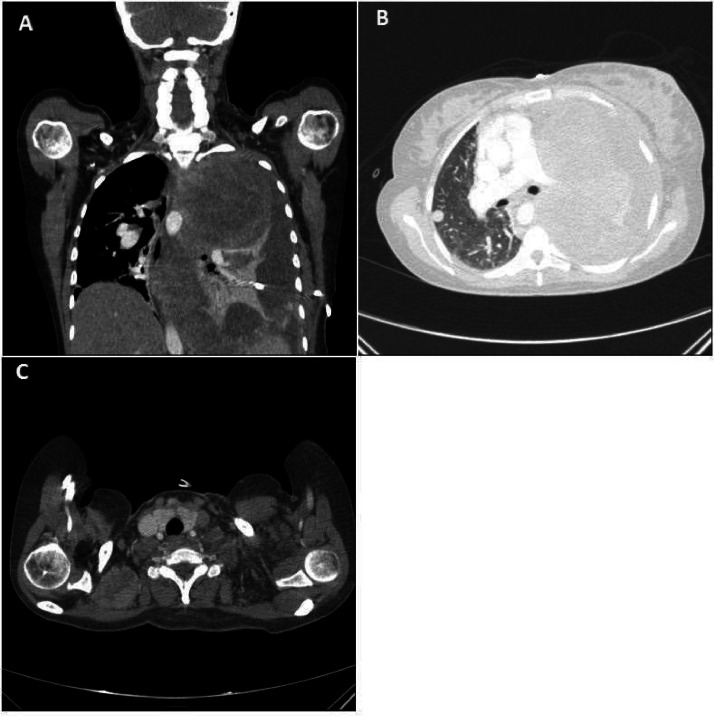
(A) Chest CT scan with IV contrast in coronal view. (B) Evidence of a large heterogeneous lobulated enhancing mass is seen in the left side of the mediastinum with extension to the left upper and lower lobes. Spiral chest CT scan in axial view showed a round pulmonary nodule in the lateral aspect of the right upper lobe. (C) Neck CT Scan Axial view showed heterogeneously enhancing soft tissue density lymph node in the supraclavicular region.

For further assessment of the mediastinal Mass, a tissue sample of the supraclavicular lymph node (Fig. 4) was sent to the pathology department for detailed examination, including immunohistochemistry. The following outcomes were provided: Immunohistochemistry indicated positive CD68 expression, a marker for macrophages and histiocytes. (Table 1) Based on histomorphology and immunohistochemistry, the final result was “histiocytic sarcoma”.

**Table 1:** The following outcomes were provided: Immunohistochemistry indicated positive CD68 expression, a marker for macrophages and histiocytes.

Marker	Result
C-Kit	negative
SALL4	negative
ALAP	negative
Ki67	Low (10%)
CD30	negative
LCA	negative
CD1a	negative
CD68	Positive
Cytokeratin19	negative
CD3	negative
CD20	negative
P63	negative
S100	negative
Vimentin	Positive
CD34	negative
CD117	negative
CD23	Negative

Receptor tyrosine kinase(C-Kit). Spalt-Like Transcription Factor 4(SALL4). Alpha-lipoic acid preconditioning(ALAP). Anti-Ki67(Ki67), CD30 is a transmembrane protein that is expressed in normal activated T cells, B cells, NK cells, and malignant cells. leucocyte common antigen*(*LCA*).* CD1a is a member of the lipid antigen-presenting molecules family. CD68 (Cluster of Differentiation 68) is a protein highly expressed by cells in the monocyte lineage. Cytokeratin 19 is an intermediate filament (IF) protein that is part of the Keratin family of proteins. Cluster of Differentiation 3 (CD3) is a multimeric protein complex. CD20 (encoded by the MS4A1 gene) is a B cell differentiation antigen expressed at most stages of B cell development, apart from early pro-B cells, plasmablasts, and plasma cells. Tumour protein 63 (p63) is a transcription factor of the p53 gene family. The name “S100” derives from the original isolation procedure and refers to the protein's solubility in a saturated (100%) ammonium sulfate solution of bovine brain. Vimentin is a type III intermediate filament cytoskeletal protein expressed mainly in cells of mesenchymal origin. CD34 is a unique marker for stem cell epitope and is not expressed in normal postnatal brain tissue. The CD117 protein is a transmembrane, tyrosine kinase growth factor receptor that is the product of c-kit gene expression. CD23 is an integral membrane glycoprotein involved in the regulation of IgE synthesis.

Following further evaluation for invasion and metastasis, a CT scan with intravenous contrast was conducted, confirming the presence of lung metastasis (Fig. 4).

Due to the advanced stage of the patient's HS, a committee of medical experts, including hematologists-oncologists, pathologists, pulmonologists, and obstetricians, was formed at the hospital to make a collective assessment of the case. They chose to initiate chemotherapy due to the patient's worsening condition and severe respiratory distress. Afterward, the patient received a single round of chemotherapy, which significantly improved her condition. However, despite the healthy fetus and normal obstetric sonograms, the committee decided to terminate the pregnancy.

## Patient outcome

Regrettably, despite exhaustive efforts to manage histiocytic sarcoma, the patient passed away 2 months after the first admission due to severe complications. In the final stages of her illness, the patient required intubation to address respiratory challenges. Her resilience provided valuable insights into the challenges of this aggressive malignancy. Our condolences go out to the family. The case underscores the ongoing need for advancements in understanding and treating such formidable diseases.

## Discussion

HS is an exceptionally rare hematopoietic neoplasm, and its occurrence during pregnancy adds further complexity to its diagnosis and treatment. The overall reported incidence of HS is extremely low, with only 0.17 cases per 1,000,000 individuals, underscoring its rarity [3].

Histiocytic sarcoma displays a wide spectrum of presentation and severity, ranging from localized disease to multisystem dissemination. Involvement of various anatomical sites has been previously reported, including the skin, soft tissue, gastrointestinal tract, lungs, bladder, kidneys, bones, brain, and lymph nodes, among others. This diverse range of affected systems underscores the heterogeneity of histiocytic sarcoma and highlights its potential to manifest in different anatomical locations [1,[4], [5], [6], [7], [8]].

In our presented case, the disease exhibited a particularly extensive profile, affecting various organs and systems, including the adrenal glands, lungs, multiple lymph nodes, and the development of a substantial mediastinal mass. The use of imaging tools and radiologic features can be invaluable for diagnosis, staging, and assessing the treatment's progress in such a complex disease with diverse clinical presentations and multisystem involvement.

HS presents a broad range of differential diagnoses in imaging, depending on the regions of involvement. These may include various lymphomas, such as large-cell non-Hodgkin lymphomas like DLBCL and ALCL, metastatic carcinomas, metastatic melanoma, soft tissue sarcomas, and, in some cases, infectious diseases [4,9].

Using microscopic and pathologic analysis in conjunction with an immunohistochemical marker panel is a definitive method for diagnosing within this array of differential diagnoses [2,4].

To the best of our knowledge, there have been very few reports of HS occurring during pregnancy, and we could not identify any cases similar to ours involving a mediastinal mass [10]. However, there are several reports of mediastinal masses occurring during pregnancy, which have presented with symptoms similar to our case, such as exacerbated dyspnea and tachypnea [11,12]. The crucial point to note is that dyspnea and tachypnea are frequently observed in pregnant women, and in the majority of cases, these symptoms are part of the normal physiological changes of pregnancy rather than indicating a medical issue [13,14]. However, in situations where space-occupying lesions occur in the mediastinum, the symptoms caused by the Mass and the underlying disease may mimic common pregnancy symptoms, potentially leading to a delay in diagnosis.

As a standard procedure, healthcare professionals typically do not advise any imaging procedures for patients unless there is a clear and specific medical indication. Imaging is usually suggested when there are clinical questions that demand further evaluation through diagnostic techniques, particularly when the patient is pregnant. The use of various radiologic imaging modalities in pregnant women has consistently raised concerns. Some imaging methods, such as ultrasonography, are generally considered less harmful and are specifically employed during particular times of pregnancy to monitor fetal development [15,16]. However, others are still regarded as potentially harmful to the developing fetus, with the level of risk often varying depending on the specific trimester of pregnancy. It's worth noting that magnetic resonance imaging (MRI) is generally considered safe during pregnancy, and no specific harm to the fetus is known when this modality is used [17,18]. A variety of imaging techniques utilizing ionizing radiation, such as CT scans, are employed to investigate various regions of the body, including the lungs and liver [19,20]. However, CT scans and X-rays are categorized as ionizing modalities and are not considered safe during pregnancy. Their application is limited to situations where there is a significant clinical imperative to diagnose a life-threatening condition that cannot be identified through safer imaging methods, and this necessity outweighs the possible risks to the developing fetus [16]. It is imperative to always use these modalities carefully and to minimize the ionization dose as much as possible.

Furthermore, it should be emphasized that providing the patient with comprehensive information about the condition, discussing the associated costs and benefits of available options, and obtaining the patient's informed consent are fundamental aspects of every medical process.

## Conclusion

The case of an advanced multisystem histiocytic sarcoma in a pregnant woman underscores the significant challenges in diagnosis and treatment planning. This exceptionally rare cancer presents complex issues, particularly due to its manifestation during pregnancy and its widespread impact on multiple body systems. Managing such cases demands a thorough and collaborative approach involving various medical disciplines, emphasizing the intricate coordination required in both diagnosis and treatment planning.

This case contributes to the limited literature on histiocytic sarcoma in pregnancy, emphasizing the need to consider rare malignancies in atypical presentations. It also underscores the importance of imaging in guiding clinical decisions while recognizing potential risks in pregnant patients.

## Patient consent

The authors have obtained a written informed consent from the patient to publish his case (including publication of images).
